# ePlatypus: an ecosystem for computational analysis of immunogenomics data

**DOI:** 10.1093/bioinformatics/btad553

**Published:** 2023-09-08

**Authors:** Tudor-Stefan Cotet, Andreas Agrafiotis, Victor Kreiner, Raphael Kuhn, Danielle Shlesinger, Marcos Manero-Carranza, Keywan Khodaverdi, Evgenios Kladis, Aurora Desideri Perea, Dylan Maassen-Veeters, Wiona Glänzer, Solène Massery, Lorenzo Guerci, Kai-Lin Hong, Jiami Han, Kostas Stiklioraitis, Vittoria Martinolli D’Arcy, Raphael Dizerens, Samuel Kilchenmann, Lucas Stalder, Leon Nissen, Basil Vogelsanger, Stine Anzböck, Daria Laslo, Sophie Bakker, Melinda Kondorosy, Marco Venerito, Alejandro Sanz García, Isabelle Feller, Annette Oxenius, Sai T Reddy, Alexander Yermanos

**Affiliations:** Department of Biosystems Science and Engineering, ETH Zurich, Mattenstrasse 26, Basel 4058, Switzerland; Department of Biosystems Science and Engineering, ETH Zurich, Mattenstrasse 26, Basel 4058, Switzerland; Institute of Microbiology, ETH Zurich, Vladimir-Prelog-Weg 4, Zurich 8093, Switzerland; Department of Biosystems Science and Engineering, ETH Zurich, Mattenstrasse 26, Basel 4058, Switzerland; Department of Biosystems Science and Engineering, ETH Zurich, Mattenstrasse 26, Basel 4058, Switzerland; Department of Biosystems Science and Engineering, ETH Zurich, Mattenstrasse 26, Basel 4058, Switzerland; Department of Biosystems Science and Engineering, ETH Zurich, Mattenstrasse 26, Basel 4058, Switzerland; Department of Biosystems Science and Engineering, ETH Zurich, Mattenstrasse 26, Basel 4058, Switzerland; Department of Biosystems Science and Engineering, ETH Zurich, Mattenstrasse 26, Basel 4058, Switzerland; Center for Translational Immunology, University Medical Center Utrecht, Lundlaan 6, Utrecht 3584 EA, The Netherlands; Center for Translational Immunology, University Medical Center Utrecht, Lundlaan 6, Utrecht 3584 EA, The Netherlands; Department of Biosystems Science and Engineering, ETH Zurich, Mattenstrasse 26, Basel 4058, Switzerland; Department of Biosystems Science and Engineering, ETH Zurich, Mattenstrasse 26, Basel 4058, Switzerland; Department of Biosystems Science and Engineering, ETH Zurich, Mattenstrasse 26, Basel 4058, Switzerland; Department of Biosystems Science and Engineering, ETH Zurich, Mattenstrasse 26, Basel 4058, Switzerland; Department of Biosystems Science and Engineering, ETH Zurich, Mattenstrasse 26, Basel 4058, Switzerland; Department of Biosystems Science and Engineering, ETH Zurich, Mattenstrasse 26, Basel 4058, Switzerland; Department of Biosystems Science and Engineering, ETH Zurich, Mattenstrasse 26, Basel 4058, Switzerland; Department of Biosystems Science and Engineering, ETH Zurich, Mattenstrasse 26, Basel 4058, Switzerland; Department of Biosystems Science and Engineering, ETH Zurich, Mattenstrasse 26, Basel 4058, Switzerland; Department of Biosystems Science and Engineering, ETH Zurich, Mattenstrasse 26, Basel 4058, Switzerland; Department of Biosystems Science and Engineering, ETH Zurich, Mattenstrasse 26, Basel 4058, Switzerland; Department of Biosystems Science and Engineering, ETH Zurich, Mattenstrasse 26, Basel 4058, Switzerland; Department of Biosystems Science and Engineering, ETH Zurich, Mattenstrasse 26, Basel 4058, Switzerland; Department of Biosystems Science and Engineering, ETH Zurich, Mattenstrasse 26, Basel 4058, Switzerland; Center for Translational Immunology, University Medical Center Utrecht, Lundlaan 6, Utrecht 3584 EA, The Netherlands; Department of Biosystems Science and Engineering, ETH Zurich, Mattenstrasse 26, Basel 4058, Switzerland; Department of Biosystems Science and Engineering, ETH Zurich, Mattenstrasse 26, Basel 4058, Switzerland; Department of Biosystems Science and Engineering, ETH Zurich, Mattenstrasse 26, Basel 4058, Switzerland; Department of Biosystems Science and Engineering, ETH Zurich, Mattenstrasse 26, Basel 4058, Switzerland; Institute of Microbiology, ETH Zurich, Vladimir-Prelog-Weg 4, Zurich 8093, Switzerland; Department of Biosystems Science and Engineering, ETH Zurich, Mattenstrasse 26, Basel 4058, Switzerland; Department of Biosystems Science and Engineering, ETH Zurich, Mattenstrasse 26, Basel 4058, Switzerland; Institute of Microbiology, ETH Zurich, Vladimir-Prelog-Weg 4, Zurich 8093, Switzerland; Center for Translational Immunology, University Medical Center Utrecht, Lundlaan 6, Utrecht 3584 EA, The Netherlands; Department of Pathology and Immunology, University of Geneva, 24 rue du Général-Dufour, Geneva 1211, Switzerland

## Abstract

**Motivation:**

The maturation of systems immunology methodologies requires novel and transparent computational frameworks capable of integrating diverse data modalities in a reproducible manner.

**Results:**

Here, we present the ePlatypus computational immunology ecosystem for immunogenomics data analysis, with a focus on adaptive immune repertoires and single-cell sequencing. ePlatypus is an open-source web-based platform and provides programming tutorials and an integrative database that helps elucidate signatures of B and T cell clonal selection. Furthermore, the ecosystem links novel and established bioinformatics pipelines relevant for single-cell immune repertoires and other aspects of computational immunology such as predicting ligand–receptor interactions, structural modeling, simulations, machine learning, graph theory, pseudotime, spatial transcriptomics, and phylogenetics. The ePlatypus ecosystem helps extract deeper insight in computational immunology and immunogenomics and promote open science.

**Availability and implementation:**

Platypus code used in this manuscript can be found at github.com/alexyermanos/Platypus.

## 1 Introduction

The fields of systems and computational immunology have advanced substantially in recent years, most notably through progress in genomics and single-cell sequencing, which are transforming the measurement of adaptive immune responses from qualitative to quantitative science. In recent years, a number of bioinformatic software tools have been developed that provide rapid and facile exploration of single-cell RNA sequencing (scSeq) data and perform analyses such as differential gene expression, cell clustering and transcriptional phenotyping (Satija *et al.* 2015, Efremova *et al.* 2020). However, in the context of immunogenomics, lymphocytes (B and T cells) and their transcriptomes and immune receptor repertoires (B cell receptor, BCR and T cell receptor, TCR), there is a lack of software enabling the simultaneous interrogation and integration of multiple approaches capable of deconstructing high-dimensional immune responses, such as phylogenetics, machine learning, graph theory, and structural modeling. Moreover, although deep sequencing of immune repertoires has become a common method in modern immunology, locating, downloading, and integrating data across experiments and research groups remains challenging. Finally, most immunogenomics software tools require computational expertise involved in analyzing such feature-rich datasets (Yaari and Kleinstein 2015, Yermanos *et al.* 2017, Borcherding *et al.* 2020).

## 2 Ecosystem overview

**Figure 1.**
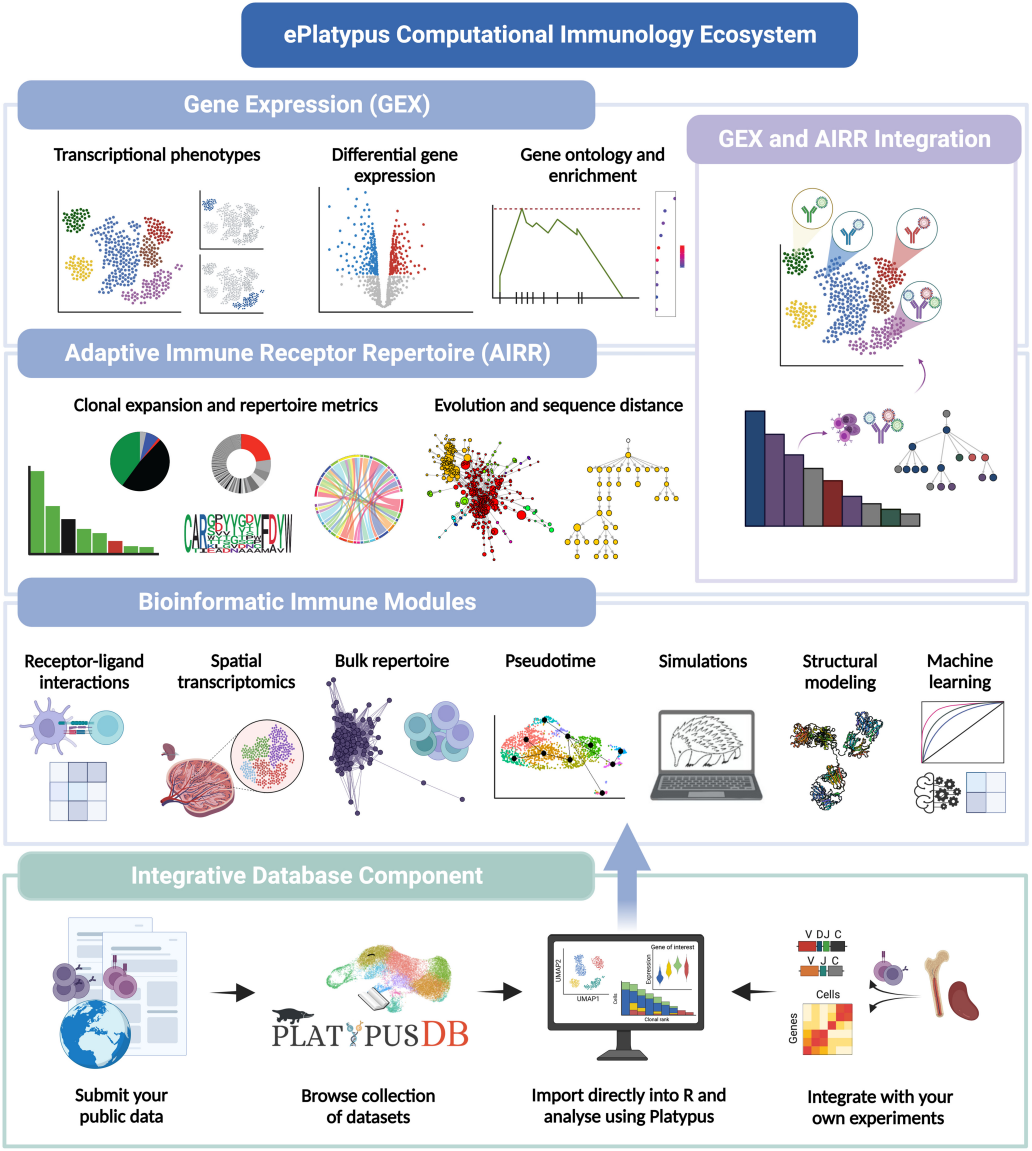
Breadth of the ePlatypus computational immunology ecosystem. The ecosystem currently is composed of a core R package that has pipelines pertaining to immune repertoires, gene expression, receptor–ligand interactions, spatial transcriptomics, pseudotime, simulations, structural modeling, and machine learning. Similarly, the ecosystem contains an integrated database and a website currently containing 21 tutorials with accompanying code.

Here, we present ePlatypus, a computational immunology ecosystem that expands upon Platypus (Yermanos *et al.* 2021a), a previously developed immunogenomics software. The ePlatypus ecosystem (Fig. 1) consists of hundreds of R and python functions, including those most relevant for single-cell immunogenomics (transcriptomes and immune repertoires) as well as many other aspects of computational immunology. More specifically, this novel ecosystem represents a complete rework from the original Platypus R package (Yermanos et al. 2021a), and has been rebuilt around a central data object that is now compatible with R and python and can directly store and integrate features such as gene expression, immune receptors, spatial coordinates, and structural information (Supplementary Table S1). This central object can be directly supplied as input for novel downstream applications and modules spanning a wide-range of immunogenomics and bioinformatics applications (Supplementary Tables S1 and S2). Additionally, the ePlatypus ecosystem contains a database component, PlatypusDB, that directly integrates into the R programming language, thereby allowing the rapid analysis and integration of B and T cells containing both adaptive immune receptor information (VDJ) and single-cell transcriptomes (GEX). PlatypusDB both stores raw output files from the commonly used aligner tool Cellranger (10x Genomics) and also holds the immune-relevant data in the form of an R object that can be loaded directly into the R environment without explicitly requiring file download. Importantly, the data is stored as both the processed aligned output and as a preprocessed R object that contains transcriptome, immune repertoire, and metadata information. Within the programming interface, the user has the ability to perform the following actions: (i) download entire public sequencing datasets, (ii) download individual samples from publications, and (iii) download and integrate public repertoires with samples stored locally (Fig. 1). While the ePlatypus development team will continuously update the ecosystem with newly published datasets, external users can also submit their preprocessed immune receptor repertoires directly for manual curation and addition to the database.

## 3 Usage and application

To demonstrate several use cases of the ePlatypus computational ecosystem, we integrated and analyzed multiple single-cell transcriptomes and immune receptor repertoires across different disease conditions, viral infections, and vaccination studies (Supplementary Fig. S1 and Supplementary Table S3). These datasets were used to highlight various modules including: (i) pseudobulking differential expression pipelines to robustly characterize transcriptional clusters leveraging methods originally designed for bulk RNA-sequencing (Supplementary Fig. S2), (ii) immune repertoire diversity metrics to characterize clonal distributions and to ensure sufficient sampling depths have been recovered (Supplementary Fig. S3), (iii) phylogenetics to identify evolutionary trajectories and intraclonal network properties of B cells during infection (Supplementary Fig. S4), (iv) B and T cell sequence similarity networks to identify fundamental principles of lymphocyte repertoire architecture in the course of an immune response (Supplementary Fig. S4), (v) machine-learning guided classification to predict BCR and TCR specificity and further uncover feature importance of antigen-specific sequences (Supplementary Fig. S5), (vi) predicting ligand–receptor interactions under homeostatic and disease conditions using the CellphoneDB repository (Efremova *et al.* 2020) (Supplementary Fig. S6), (vii) spatial transcriptomics to spatially interrogate gene expression patterns and further integrate clonal selection and clonal evolution of adaptive immune responses (Supplementary Fig. S7), and (viii) structural modeling of immune receptor sequences and repertoires with the Steropodon pipeline using multiple external tools including AlphaFold, IgFold, and DeepAb (Jumper *et al.* 2021) (Supplementary Fig. S8). Furthermore, ePlatypus now supports python functionality for the implementation of repertoire analyses such as investigating clonal expansion and isotype distribution. This python pipeline can also be supplied to more advanced machine learning and artificial intelligence workflows such as the use of protein language models, including both foundational and receptor-specific language models such as ProtBERT, Sapiens (Prihoda *et al.* 2022), AntiBERTy (Ruffolo *et al.* 2021), ESM-1B (Lin *et al.* 2023), Ablang (Olsen *et al.* 2022), and TCR-BERT (Wu *et al.* 2021) for repertoire feature visualization and classification (Supplementary Fig. S9). Importantly, ePlatypus currently hosts an online portal with 21 educational tutorials and walk-throughs (Supplementary Fig. S10), each of which contain code, comments, and explanatory text (Supplementary Fig. S11) for various computational immunology frameworks (Supplementary Table S2).

To further demonstrate several use cases of the ePlatypus computational ecosystem and accompanying database, we integrated and analyzed multiple single-cell transcriptomes and immune receptor repertoires across different disease conditions, viral infections, and vaccination studies (Supplementary Fig. S1 and Supplementary Table S3). We directly downloaded murine T cell repertoires from previously published datasets containing both CD4 and CD8 T cells from conditions such as acute and chronic viral infections (Khatun *et al.* 2021, Merkenschlager *et al.* 2021, Kuhn *et al.* 2022, Shlesinger *et al.* 2022), homeostatic aging (Yermanos *et al.* 2021b), and experimental autoimmune encephalomyelitis (Shlesinger *et al.* 2022) (Supplementary Fig. S1 and Supplementary Table S3). Following transcriptional integration with Harmony (Korsunsky *et al.* 2019), which aims to reduce batch effects across different datasets, we visualized all cells using uniform manifold approximation projection (UMAP) (Supplementary Fig. S12A). This demonstrated two major transcriptional regions, dominated by either Cd4 or Cd8 gene expression, which could be simultaneously interrogated with other known gene markers of activation or exhaustion such as Cd44, Ifng, Pdcd1, Lag3, and Il7r (Supplementary Fig. S12B). Supplementing this focused analysis with ProjectTILS, a recently developed reference atlas which helps resolve murine T cell heterogeneity of tumor-infiltrating T cells (Andreatta *et al.* 2021), demonstrated that T cells from PlatypusDB almost entirely cover the ProjecTILs main reference dataset (Supplementary Figs S12C–E and S13).

Next, we explored whether transcriptional heterogeneity could similarly be detected for B cells present in PlatypusDB. Multiple datasets derived from murine models of infection, immunization, and autoimmune disease (Merkenschlager *et al.* 2021, Yewdell *et al.* 2021, Neumeier *et al.* 2022, Shlesinger *et al.* 2022, Agrafiotis *et al.* 2023) were integrated as previously performed with T cells (Supplementary Fig. S1 and Supplementary Table S3). Transcriptional analysis using both canonical B cell markers and previously reported B cell gene signatures highlighted the presence of diverse B cell subtypes present in PlatypusDB across multiple datasets (Supplementary Fig. S14A and B). For example, our database contains a large number of ASCs, identified based on expression of Sdc1 (Cd138), Xbp1, and Slamf7, which exhibited varying expression levels of markers relating to chemokine receptors (Cxcr3 and Cxcr4) and B cell proliferation (Mki67) (Supplementary Fig. S14C).

## 4 Concluding remarks

The analyses presented here highlight the breadth of B and T cell phenotypes and selection patterns already available within ePlatypus, which will only continue to grow as more user-supplied public datasets are added. Lastly, we computed the runtime of several pipelines within the ePlatypus ecosystem on datasets of varying size and cell numbers, highlighting the scalability and speed of our software (Supplementary Table S4).

The maturation of systems immunology methodologies requires novel and transparent computational frameworks capable of integrating diverse data modalities in a reproducible manner. The ePlatypus ecosystem, composed of hundreds of R and python functions, programming tutorials, and a comprehensive database, helps extract deeper insight in immunogenomics while promoting open science.

## Supplementary Material

btad553_Supplementary_Data

## Data Availability

The accession numbers and publications for the sequencing data used in this manuscript are located in Supplementary Table S1. Platypus code used in this manuscript can be found at github.com/alexyermanos/Platypus.
