# Supplementary material for: ePlatypus: an ecosystem for computational analysis of immunogenomics data
Source: Bioinformatics. 2023 Sep 8;39(9):btad553. doi: 10.1093/bioinformatics/btad553 (PMC10518073; doi:10.1093/bioinformatics/btad553)
Supplement: btad553_Supplementary_Data [file btad553_supplementary_data.zip › ePlatypus_online_methods.pdf]

ePlatypus: an ecosystem for computational analysis of immunogenomics data

Online Methods

PlatypusDB architecture: The computational infrastructure for PlatypusDB was developed based on the analysis package Platypus and the Google Cloud storage API. The process of uploading a dataset includes the following steps: First, raw sequencing files are sourced locally, downloaded from public repositories such as GEO, or acquired directly from another research group. Raw reads were then aligned with Cellranger 6.0.1 to the following 10x Genomics reference genomes: refdata-gex-mm10-2020-A, refdata-cellranger-vdj-GRCm38-alts-ensembl-5.0.0, refdata-gex-GRCh38-2020-A, and refdata-cellranger-vdj-GRCh38-alts-ensembl-5.0.0. Raw output files were then uploaded as compressed directories to the PlatypusDB Google Cloud storage database. Raw outputs were then loaded and processed in R resulting in two formats. Firstly a per-sample list object containing main Cellranger output tables and secondly a VDJ-GEX-matrix object from Platypus v3.5.0. This object was generated using the VDJ_GEX_matrix function with default settings, if not otherwise noted. All output objects were uploaded to PlatypusDB using the package googleCloudStorageR. To allow for easy access to the database, download of R objects as well as compressed directories is available directly via URL without the need to install Google Cloud storage compatibility packages for R. The URL for a database lookup table is delivered with Platypus and allows for a single access point to the database, which remains constant as more datasets will be added in the future.

Data analysis: The filtered feature matrix directory was supplied as input to the VDJ_GEX_matrix function in the R package Platypus (v3.5.0), which uses the transcriptome analysis workflow of the R package Seurat (Satija et al. 2015). Only those cells containing less than 20% of mitochondrial reads were retained in the analysis. Genes involved in the adaptive immune receptor (e.g., TRB, TRBV1-1),

were removed from the count matrix to prevent clonal relationships from influencing transcriptional phenotypes. Gene expression was normalized using the “harmony” argument in the VDJ_GEX_matrix function. 2000 variable features were selected using the “vst” selection method and used as input to principal component analysis (PCA) using the first 10 dimensions. Graph-based clustering using the Louvain modularity optimization and hierarchical clustering was performed using the functions FindNeighbors and FindClusters in Seurat using the first ten dimensions and a cluster resolution of 0.5. UMAP was similarly inferred using the first ten dimensions. The FindMarkers function from Seurat was used when calculating differentially expressed genes (both across groups or across clusters) with logfc.threshold set to 0 and minimum number of cells expressing each gene set to 0.25 and subsequently supplied to the GEX_volcano function from Platypus. Mitochondrial and ribosomal genes were removed when visualizing DE genes. Feature plots were produced by supplying genes of interest to the function FeaturePlot in Seurat. Module scores for public gene sets (Mathew *et al.* 2021) were calculated using the AddModuleScore from Seurat. Cells containing no or more than one α/heavy and β/light chain were filtered out for TCR/BCR repertoire analysis. Clones were defined by identical CDR3α/CDRH3 and CDR3β/CDRL3 sequence (nucleotide or amino acid sequence) across all repertoires. Clones represented by more than one cell were considered highly-expanded clones, while single-celled clones were defined as lowly-expanded. The projection of cells onto reference UMAPs and cell state predictions were done using the R package ProjecTILs (Andreatta et al. 2021) under default conditions. Experiments were either individually or all together projected onto the ProjecTILs atlas. Briefly, the input to ProjecTILs is a query dataset in the form of a gene expression matrix. The gene expression matrix is aligned to the reference map, to bring the query data into the same scale as the reference map. The UMAP transformation calculated on the reference map is applied to the query set, effectively embedding it into the same space of the reference map, and allowing their direct comparison and joint visualization (Andreatta *et al.* 2021). For Figures S1 to S8, single-cell immune repertoire sequencing experiments present in PlatypusDB were formatted into a

single VDJ_GEX_matrix object that was then supplied to downstream analyses pipelines. Specifically, the pseudobulk analysis was performed using the pseudo_bulk_DE function from Platypus. Sampling and diversity analyses were performed using the VDJ_diversity and VDJ_rarefaction. The sequence similarity network of clusters was generated using the AntibodyForests_communities function and then colored using the AntibodyForests_plot. Node and edge metrics were calculated using the AntibodyForests_metrics function from Platypus. The PlatypusML_classification function from Platypus, which takes input the encoded features obtained from the PlatypusML_extract_features function, was used to run cross validation on a specified number of folds for different classification models (XGBoost, SVM, Random Forest, Logistic Regression & Gaussian Naive Bayes), outputting the AUC scores, ROC curve and confusion matrix for each classification model. PLM analyses were performed by supplying the heavy chain amino-acid sequences from 1500 clonotypes as input to the PLM pipeline using ProtBERT (Brandes *et al.* 2022). The embeddings were extracted using the last layer, CLS token, and were visualized using UMAP with 15 nearest neighbors. Prediction of receptor-ligand interaction was calculated on the single-cell gene expression data using the CellPhoneDB software. The heatmap and dot plot were generated as an output of the CellPhoneDB_analyse function from Platypus. Receptor structures in the Steropodon workflow were obtained using the Steropodon_model function and visualized using the Steropodon_visualize function in Platypus. Computational performance of several functions from R package Platypus (v3.5.0) can be seen in Table S4. Performance was calculated using a single cell sequencing sample with 5914 GEX and 5200 VDJ cell barcodes. The same sample was used to showcase performance for datasets of n=5 and n=20 sample size.

Data visualization: Figure 1 and the supplementary graphical overviews were created with Biorender.com. Feature plots were produced using “FeaturePlot” (Seurat 4.0). Volcano plots were produced using “GEX_volcano” (Platypus v3.5.0). Dottile plots were produced using DotPlot (Seurat 4.0). All other figures were produced using Prism v9 (Graphpad).

Data availability: The accession numbers and publications for the sequencing data used in this manuscript are located in table S1. Platypus code used in this manuscript can be found at github.com/alexyermanos/Platypus.
